# Compromised longevity due to *Mycobacterium abscessus* pulmonary disease in lungs scarred by tuberculosis

**DOI:** 10.1099/acmi.0.000003

**Published:** 2019-03-20

**Authors:** Urvashi B. Singh, Rojaleen Das, Prajowl Shrestha, Kiran Bala, Pooja Pandey, Santosh Kumar Verma, Hitender Gautam, Elizabeth Story-Roller, Gyanu Lamichhane, Randeep Guleria

**Affiliations:** 1 All India Institute of Medical Sciences, Department of Microbiology, New Delhi, India; 2 All India Institute of Medical Sciences, Department of Pulmonary Medicine and Sleep Disorders, New Delhi, India; 3 Division of Infectious Diseases, School of Medicine, Johns Hopkins University, Baltimore, Maryland, USA

**Keywords:** *M. abscessus*, faropenem, pulmonary disease, tuberculosis

## Abstract

Structural lung diseases or scarring related to prior infections such as tuberculosis (TB) are risk factors for the development of invasive nontuberculous mycobacterial (NTM) pulmonary infections, such as *Mycobacterium abscessus . M. abscessus* is intrinsically resistant to many antibiotics and *in vitro* susceptibility correlates poorly with clinical response, especially in pulmonary disease. Treatment is often difﬁcult due to the lack of effective antibiotic regimens. We present a case of a 56-year-old male previously treated for TB, with presumed exacerbation, who was diagnosed after much delay with pulmonary *M. abscessus* disease and subsequently failed initial treatment with an empirical antibiotic regimen. When placed on a synergistic combination regimen that included amikacin, linezolid, clarithromycin, ethambutol and faropenem, the patient showed a favourable response and was culture-negative for over 12 months when the treatment was stopped as per American Thoracic Society (ATS) recommendations. Unfortunately, he developed recurrent symptoms and died 9 months after stopping treatment, following an acute exacerbation of fever and respiratory failure.

## Case

**Fig. 1.**
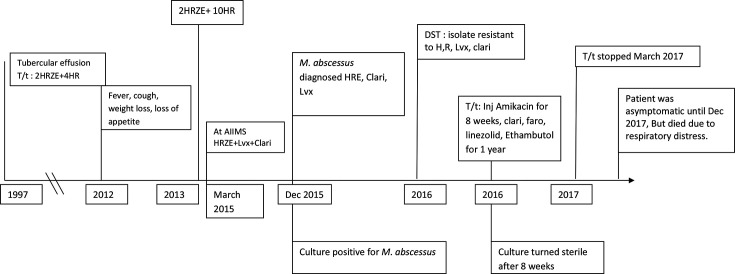
Time line of events. H, isoniazid; R, rifampicin; Z, pyrazinamide; E, ethambutol; Lvx, levofloxacin; Clari, clarithromycin; Faro, faropenem; DST, drug susceptibility testing.

A 56-year-old non-smoking male, retired army personnel, presented to the pulmonary outpatient clinic with symptoms of fever, productive cough and weight loss in March 2015 at AIIMS, New Delhi. He had initially presented with symptoms of fever, expectoration, weight loss and loss of appetite in 2012. He was given empirical anti-tubercular treatment for a clinical diagnosis of tuberculosis for 13 consecutive months with no improvement in 2013. The patient had been treated for pleural effusion in 1997, and declared cured. In March 2015 the patient reported to our institute. Examination revealed bilateral bronchial breath sounds in the upper and lower chest regions. His sputum samples were acid-fast bacilli (AFB)-positive and indicated suspected relapse of tuberculosis (TB); treatment was initiated with six drugs, including rifampicin, isoniazid, ethambutol, clarithromycin, levofloxacin and linezolid with a supplement of vitamin B6. However, his symptoms worsened (Fig. 1).

## Investigations

**Fig. 2.**
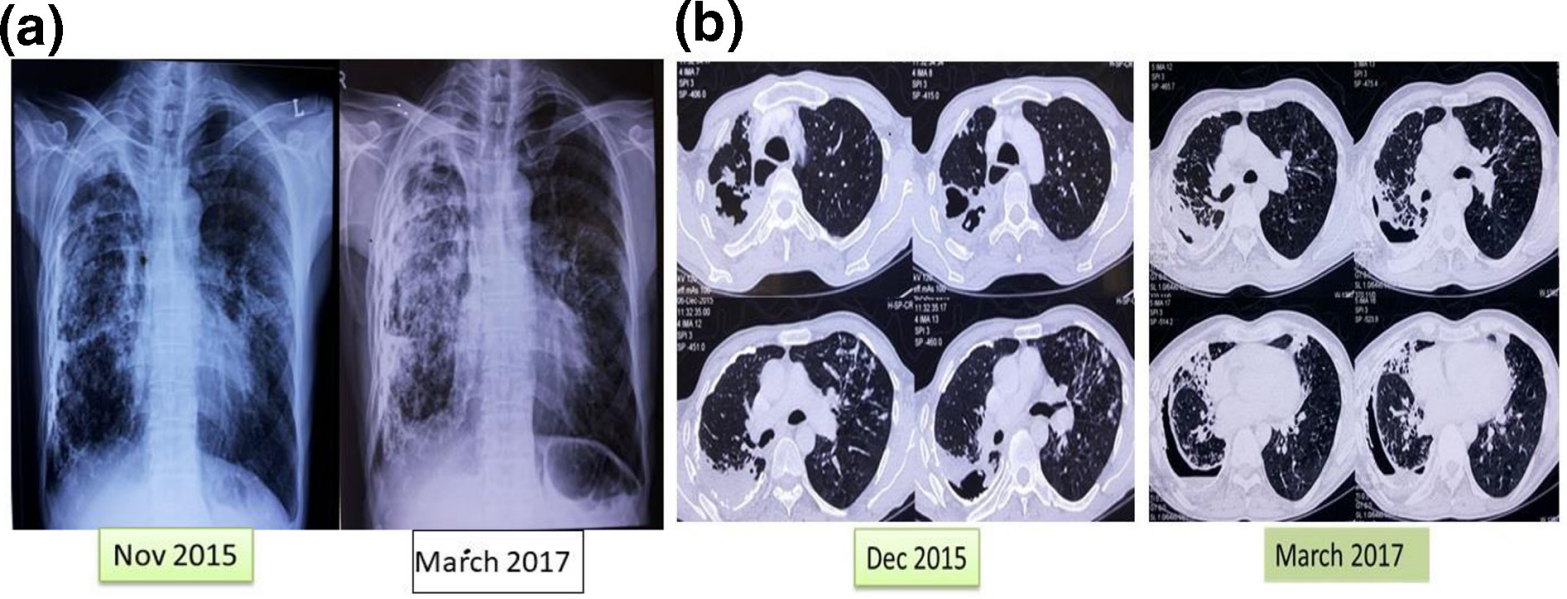
(a) Chest X-ray PA view (November 2015) shows pleural thickening and calcification of right lung with multiple scattered lung nodules and cystic lucencies ion the right side of the lung. Chest X-ray (March 2017) shows pleural thickening and calcification of right lung with multiple scattered lung nodules and cystic lucencies ion the right side of the lung. There is resolution of left middle zone lung infiltrates. Compensatory hyperinflation of left lung is noted. (b) Chest computed tomography (CT) (December 2015) showed right upper lobe thick-walled cavity with irregular internal wall. There are areas of centrilobular nodules surrounding the cavity and left upper lobe, suggesting an active infective process. However, a chest CT in March 2017 showed that the centrilobular nodules in the left lower lobe had decreased as compared to those in the previous one.

A complete blood count showed elevated lymphocyte and monocyte counts of 14.9 * 10^3^ µl^−1^ and 8.9 * 10^3^ µl^−1^, respectively, as well as an elevated erythrocyte sedimentation rate (ESR) of 40 mm after 1 h. Blood chemistry findings were normal. The patient was non-reactive for HIV 1 and 2 as well as hepatitis B and C. The workup for other bacteria and fungi was negative. A chest X-ray showed pleural thickening with hydropneumothorax and bilateral fibrous parenchymal lesions (Fig. 2). Sputum microscopy was positive with a Revised National Tuberculosis Control Program grading of (1+). A Gene Xpert assay was performed on the sample and was found to be negative. Culture on Lowenstein–Jensen (LJ) medium and Mycobacteria Growth Indicator Tube (MGIT 960) grew short, slender and curved AFB within 5 and 3 days, respectively, indicating a rapidly growing organism. An MGIT TBc identification test (Becton Dickinson) was negative and other bacteria were ruled out by culture. Subculture on LJ medium showed buff and pasty colonies within 5 days, indicating a rapid grower. The isolate was negative for a niacin accumulation test, a heat-stable catalase test and a nitrate reductase test, while it was NaCl-tolerant and grew on MacConkey’s medium. Hence, it was identified as *Mycobacterium abscessus *. A second sputum sample was also identified as *M. abscessus *. Hence, *M. abscessus* was clearly the pathogen in this case scenario (ATS, American Thoracic Society guidelines) [1]. The isolate was subsequently sequenced for the 16S ribosomal gene and confirmed as *M. abscessus *, and the sequence was submitted to GenBank and assigned accession no. MF287216.

*M. abscessus* is intrinsically resistant to many antibiotics and *in vitro* susceptibility correlates poorly with clinical response, especially in pulmonary disease [2–4]. Drug susceptibility testing on the isolate demonstrated resistance to amikacin, imipenem, moxifloxacin and linezolid based on established minimum inhibitory concentration (MIC) breakpoints [5]. Of note, the isolate was susceptible to clarithromycin. Several studies have demonstrated synergy between antibiotics against *M. abscessus in vitro*, which has the potential to decrease the MIC of both drugs and overcome antibiotic resistance. Synergy between linezolid and clarithromycin [6, 7] and linezolid and amikacin [8], as well as several carbapenems and rifampicin [9], have been described. Additionally, although the MIC of ethambutol was not specifically tested and many *M. abscessus* isolates tend to harbour inherent resistance to ethambutol [10], cases of ethambutol inclusion in treatment regimens against *M. abscessus* have been documented [11]. Given the extensive resistance profile of this isolate, the potential for synergy of the above-listed drugs in combination with faropenem was tested (Table 1). The MIC results from these tests, in addition to the synergy studies cited above, were used to develop an antibacterial treatment regimen for the patient. Adjunctive surgical resection unfortunately could not be considered in this case due to the involvement of more than half of the right lung.

**Table 1.** Synergistic effect on MIC from the addition of faropenem to rifampicin, amikacin, moxifloxacin and linezolid. Clinical and Laboratory Standards Institute drug susceptibility testing breakpoints for *M. abscessus *: rifampicin (not established), imipenem (MIC≥16 µg ml^−1^), amikacin (MIC≥64 µg ml^−1^), linezolid (MIC≥32 µg ml^−1^) and moxifloxacin (MIC≥4 µg ml^−1^). Faropenem tested at 0.625 µg ml^−1^ in synergy studies

**Drug name**	**Drug concentrations**				
	10 µg ml^−1^	5 µg ml^−1^	2.5 µg ml^−1^	1.25 µg ml^−1^	0.625 µg ml^−1^
Faropenem only	Resistant	Resistant	Resistant	Resistant	Resistant
	1.0 µg ml^−1^	0.5 µg ml^−1^	0.25 µg ml^−1^	0.12 µg ml^−1^	0.063 µg ml^−1^
Rifampicin only	Resistant	Resistant	Resistant	Resistant	Resistant
Rifampicin and faropenem	Susceptible	Susceptible	Susceptible	Susceptible	Susceptible
	64 µg ml^−1^	32 µg ml^−1^	16 µg ml^−1^	8 µg ml^−1^	4 µg ml^−1^
Amikacin only	Susceptible	Resistant	Resistant	Resistant	Resistant
Amikacin and faropenem	Susceptible	Susceptible	Susceptible	Resistant	Resistant
	128 µg ml^−1^	64 µg ml^−1^	32 µg ml^−1^	16 µg ml^−1^	8 µg ml^−1^
Linezolid only	Susceptible	Resistant	Resistant	Resistant	Resistant
Linezolid and faropenem	Susceptible	Resistant	Resistant	Resistant	Resistant
	4 µg ml^−1^	2 µg ml^−1^	1 µg ml^−1^	0.5 µg ml^−1^	0.25 µg ml^−1^
Moxifloxacin only	Susceptible	Susceptible	Resistant	Resistant	Resistant
Moxifloxacin and faropenem	Susceptible	Susceptible	Susceptible	Resistant	Resistant

µg ml^−1^, microgram per millilitre.

Given the reported disconnect between the *in vitro* susceptibility of *M. abscessus* and clinical response [4], the decision was made to treat the patient with linezolid (300 mg daily), clarithromycin (500 mg twice daily), ethambutol (1 g daily) and faropenem (200 mg three times daily) for 1 year, with the addition of intravenous amikacin for the initial 2-month induction phase. After 3 weeks of treatment, the patient reported improvement of symptoms, including weight gain and decreased cough. Follow-up sputum cultures converted to negative within 1 month of treatment and the patient continued on this regimen as an outpatient without any adverse effects. His condition continued to improve gradually and he became persistently culture-negative for 12 months after starting treatment, at which time antibiotics were stopped as per American Thoracic Society (ATS) recommendations [1]. The patient remained asymptomatic for the next 9 months, but subsequently developed recurrence of respiratory symptoms. He unfortunately succumbed to an acute exacerbation of fever and respiratory failure before he could report to our institute.

## Discussion

Prior pulmonary TB and bronchiectasis are predisposing conditions for *M. abscessus* pulmonary infection and most patients with lung disease due to *M. abscessus* are more than 60 years of age [1]. The disease usually presents with symptoms that are similar to those of other respiratory pathogens, including cough and easy fatigability, and it is difficult to differentiate from TB by radiography. *M. abscessus* is intrinsically resistant to several classes of antibiotics and is known to produce poor clinical response even to agents showing *in vitro* susceptibility. This is especially true in the setting of pulmonary disease [1]. Current guidelines recommend prolonged multidrug therapy to minimize the chances of relapse [1, 12]. Chronic pulmonary disease due to nontuberculous mycobacteria (NTM) is difficult to treat and rarely cured [13].

According to the ATS guidelines, there are no regimens of proven efficacy for treating pulmonary disease caused by *M. abscessus *. The ATS recommends a clarithromycin-based multidrug regimen, along with surgical resection of localized disease. However, not all subspecies of *M. abscessus* are susceptible to clarithromycin. Recently, inducible resistance to clarithromycin has been described in *M. abscessus* isolates on extended incubation *in vitro* and the *erm* gene confers naturally inducible resistance *in vivo*. The production of Erm (41) methyltransferase predicts clinical failure for clarithromycin [14]. This further complicates the development of adequate treatment regimens.

As stated above, several studies have demonstrated the synergistic activity of various classes of antibiotics against *M. abscessus *. Emerging evidence suggests that the carbapenem and penem classes of *β*-lactams are potent against *M. abscessus* [15, 16]. Additionally, there is a case report of successful treatment of *M. abscessus* pulmonary disease with a combination of faropenem and clarithromycin [17]. Hence, faropenem, a new, orally bioavailable penem, which exhibits a broad spectrum of activity against Gram-negative, Gram-positive and anaerobic bacteria [18], as well as synergistic efficacy against *M. abscessus* [9], was included in the regimen.

In the present case, *in vitro* synergy was demonstrated against the isolate, as the addition of faropenem reduced the MIC of rifampicin by 16-fold, that of amikacin by 4-fold, and that of moxifloxacin by 2-fold, although no change was seen in the MIC of linezolid with the addition of faropenem (Table 1). Based on these studies, a multidrug treatment regimen was designed that included 2 months of intravenous (IV) amikacin during the initial induction phase of treatment, combined with oral linezolid, clarithromycin, ethambutol and faropenem, which were continued for 1 year, although the isolate was resistant to amikacin, linezolid and faropenem individually *in vitro*. The patient showed rapid clinical and microbiological improvement within a month of treatment initiation. Subsequently, the patient continued to have negative sputum cultures throughout the duration of treatment, even after the discontinuation of IV amikacin.

This novel oral regimen of linezolid, clarithromycin, ethambutol and faropenem contributed to successful treatment of this patient’s infection, as evidenced by the clinical resolution of symptoms and maintenance of culture negativity for 12 months. It is interesting to note that this patient had initially been considered to have a relapsed or recurrent TB infection upon presentation to our institute and was hence treated with rifampicin, isoniazid, ethambutol, clarithromycin, levofloxacin and linezolid; however, his symptoms persisted. Therefore, it may be reasonable to assume that the addition of faropenem to his *M. abscessus* treatment regimen significantly increased the efficacy of the regimen, as the prior empirical regimen containing the other three oral antibiotics (linezolid, clarithromycin and ethambutol) had not improved his symptoms. The use of IV amikacin during the induction phase of treatment likely contributed to his rapid sputum culture conversion, but also exhibited synergy with faropenem, potentially making it more effective.

Subsequently, the patient remained asymptomatic for few months, having assumed that he was cured. Unfortunately, 9 months after stopping therapy the patient developed recurrence of symptoms, which ultimately led to respiratory failure and death, presumably due to relapse of infection. Ideally, post-mortem analysis would have been helpful to confirm relapsed *M. abscessus* infection as the cause of death, but this was not feasible (Fig. S1, available in the online version of this article).

This case emphasizes how difficult it is to achieve an effective cure in the setting of *M. abscessus* pulmonary disease, which was also noted in a recent systematic review that reported relapse-free sputum culture conversion rates of as low as 25 % despite prolonged multidrug therapy [19]. This case also highlights the importance of close medical follow-up in these patients even after the completion of treatment, given the high rate of relapse.

### Conclusion

Early diagnosis of *M. abscessus* pulmonary disease is imperative for the initiation of appropriate treatment and the prevention of further damage to infected tissues. This patient likely developed *M. abscessus* lung disease as a result of his prior TB infection, which caused lung scarring and bronchiectasis, and was further exacerbated by delayed diagnosis of a nontuberculous mycobacterial infection. *In vitro* susceptibility to antimicrobial agents does not always correlate with clinical response in pulmonary disease due to *M. abscessus *, which may hinder the development of appropriate treatment regimens. Several studies have reported synergy among various classes of antibiotics against *M. abscessus *, which have the potential to overcome drug resistance when used in combination. Although this patient ultimately succumbed to a presumed relapse of infection 9 months after the cessation of antibiotics, his novel multidrug regimen resulted in persistent sputum culture negativity during the 12 months of treatment, which indicates treatment success per the ATS guidelines. This case describes the successful use of a novel synergistic antibiotic regimen against a highly drug-resistant strain of *M. abscessus *. It also emphasizes the importance of close medical follow-up after the cessation of treatment and early intervention in the setting of relapsed disease.

## Supplementary Data

Supplementary material 1
