# Supplementary material for: Compromised longevity due to Mycobacterium abscessus pulmonary disease in lungs scarred by tuberculosis
Source: Access Microbiol. 2019 Mar 20;1(1):e000003. doi: 10.1099/acmi.0.000003 (PMC7470354; doi:10.1099/acmi.0.000003)
Supplement: Supplementary material 1 [file acmi-1-003-s001.pdf]

Probable cause of death (As Discussed with his Daughter)

Patient was doing well till December 2017. From the second week of December 2017, he developed swelling of bilateral lower limb and respiratory distress along with fever. He was initially admitted and evaluated in Jabalpur (His native place). He was managed with oxygen supplementation and IV antibiotics. He was then brought to AIIMS, New Delhi for further evaluation. he was admitted and same treatments were continued. During hospital stay he developed Type II respiratory failure which was managed with bronchodilators and BiPAP. He improved with the treatment and after 12 days of admission, he was discharged. He went back to Jabalpur, and 2 days after reaching there he developed altered sensorium, he was taken to nearest hospital, where he was found to have hyponatremia. He was given IV normal saline for Hyponatremia and subsequently discharged. While on his way to back home in ambulance, his daughter found him unconscious, he was again taken back to hospital, where he was declared dead.

(According his daughter, at the time of discharge he was fine and was conscious. But on the way back to home he suddenly became unconscious. He was rushed back to same hospital, where he was declared dead. Reviewing the history, it seems that he must had some cardiac event, which led to sudden cardiac death.)
